# Chronic Hepatitis C Virus Infection Impairs M1 Macrophage Differentiation and Contributes to CD8^+^ T-Cell Dysfunction

**DOI:** 10.3390/cells8040374

**Published:** 2019-04-25

**Authors:** Faria Ahmed, Andrea Ibrahim, Curtis L. Cooper, Ashok Kumar, Angela M. Crawley

**Affiliations:** 1 Department of Biochemistry, Microbiology and Immunology, Faculty of Medicine, University of Ottawa, Ottawa, ON K1H 8M5, Canada; fahme097@uottawa.ca (F.A.); andibrahim@ohri.ca (A.I.); 2 Chronic Disease Program, Ottawa Hospital Research Institute, Ottawa, ON K1H 8L6, Canada; 3 Clinical Epidemiology Program, Ottawa Hospital Research Institute, Ottawa, ON K1H 8L6, Canada; ccooper@toh.ca; 4 Department of Medicine, Division of Infectious Diseases, The Ottawa Hospital, Ottawa, ON K1H 8L6, Canada; 5 Public Health and Preventative Medicine, School of Epidemiology, Faculty of Medicine, University of Ottawa, Ottawa, ON K1G 5Z3, Canada; 6 Department of Pathology and Laboratory Medicine, Faculty of Medicine, University of Ottawa, Ottawa, ON K1H 8M5, Canada; 7 Department of Pathology, The Children’s Hospital of Eastern Ontario Research Institute, Ottawa, ON K1H 8L1, Canada; 8 Department of Biology, Faculty of Science, Carleton University, Ottawa, ON K1S 5B6, Canada

**Keywords:** macrophages, CD8^+^ T-cells, monocyte-derived macrophages, hepatitis C virus, interferon-γ

## Abstract

Chronic hepatitis C virus (HCV) infection causes generalized CD8^+^ T cell impairment, not limited to HCV-specific CD8^+^ T-cells. Liver-infiltrating monocyte-derived macrophages (MDMs) contribute to the local micro-environment and can interact with and influence cells routinely trafficking through the liver, including CD8^+^ T-cells. MDMs can be polarized into M1 (classically activated) and M2a, M2b, and M2c (alternatively activated) phenotypes that perform pro- and anti-inflammatory functions, respectively. The impact of chronic HCV infection on MDM subset functions is not known. Our results show that M1 cells generated from chronic HCV patients acquire M2 characteristics, such as increased CD86 expression and IL-10 secretion, compared to uninfected controls. In contrast, M2 subsets from HCV-infected individuals acquired M1-like features by secreting more IL-12 and IFN-γ. The severity of liver disease was also associated with altered macrophage subset differentiation. In co-cultures with autologous CD8^+^ T-cells from controls, M1 macrophages alone significantly increased CD8^+^ T cell IFN-γ expression in a cytokine-independent and cell-contact-dependent manner. However, M1 macrophages from HCV-infected individuals significantly decreased IFN-γ expression in CD8^+^ T-cells. Therefore, altered M1 macrophage differentiation in chronic HCV infection may contribute to observed CD8^+^ T-cell dysfunction. Understanding the immunological perturbations in chronic HCV infection will lead to the identification of therapeutic targets to restore immune function in HCV^+^ individuals, and aid in the mitigation of associated negative clinical outcomes.

## 1. Introduction

Infection with hepatitis C virus (HCV) affects nearly 200 million people worldwide, establishing in most infected individuals chronicity that can lead to cirrhosis and liver cancer and has been a leading indication for liver transplantation. While new direct-acting antiviral therapies are highly effective, the effects of chronic infection on the immune system and its impact on associated clinical outcomes, such as susceptibility to unrelated infection, poor responses to vaccination, and recurrence or development of hepatocellular carcinoma (HCC), are not well-understood [1]. Chronic HCV infection has a significant impact on cells of the innate and adaptive immune system, including the shift of monocytes from anti- to pro-inflammatory states, caused in part by circulating HCV core protein [2,3,4,5,6]. The effects of impaired monocyte/macrophage function in chronic HCV infection on macrophage differentiation and subsequent influence on adaptive immune responses are not known.

Macrophages respond to their immediate environment by differentiating into pro- or anti-inflammatory cell subsets, described as M1-like or classically activated and M2-like or alternatively activated macrophages, respectively [7]. The M2 subset can be identified based on a range of markers, such as glycoproteins, scavenger receptors, enzymes, and cytokines, and are further subdivided into three sub-categories: M2a, M2b, and M2c [8]. M2a are mainly responsible for tissue repair after injury and are formed in response to IL-4, IL-13, and parasitic or fungal infections. The M2b is a regulatory subset, formed in response to immune complexes and bacterial lipopolysaccharide (LPS) stimulation; they help to dampen the effects of an immune response to control excessive inflammation. The M2c subset is formed in response to IL-10, TGF-β, and glucocorticoids [8].

The uptake of a foreign antigen by resident liver macrophages or Kupffer cells (KCs), or tissue-infiltrating macrophages, typically does not result in an inflammatory response [9]. However, signals surpassing activation thresholds can direct macrophages to produce immune activating factors, such as TNF-α, IL-6, and IL-1β [9]. Inflammatory diseases generate the pro-inflammatory cytokines interferon-γ (IFN-γ) or TNF-α, which promote M1 differentiation. In contrast, tissue homeostasis yields anti-inflammatory microenvironments with IL-4, IL-10, or IL-13 cytokines that generate anti-inflammatory M2-macrophages [10,11]. These observations confirm that soluble factors released from liver or other tissues can polarize macrophages [12].

Although studies have been limited, KC appear to be similar to monocyte-derived macrophages (MDMs) M1 (CD163^lo^, IL-12, TNF-α) or M2 (CD163^hi^CD206^hi^) subsets with pro- or anti-inflammatory effects, respectively, depending on the health status of the liver [13,14]. The study of macrophages in the liver can be challenging, particularly in HCV-infected individuals, since liver biopsy collection is no longer feasible with the use of noninvasive diagnostic monitoring of liver fibrosis as the standard of care. In addition, the origin and regeneration of KCs are thought to be from either yolk-sac-derived pre-existing cells within the liver or a continuous supply of infiltrating, bone-marrow-derived monocytes [15,16]. Nevertheless, given the known plasticity of macrophages, knowledge gained from the study of in vitro MDMs could potentially be translated to macrophages in the liver. Circulating monocytes are recruited to infected or damaged organs, such as an HCV-infected liver, where they respond to stromal-cell-derived macrophage-colony stimulating factor (M-CSF) to differentiate into macrophages to perform roles related to host defense and tissue repair [16].

By virtue of their location (lining the walls of liver sinusoids), KCs interact routinely with circulating immune cells in the blood [17]. Such interactions, including that of infiltrating macrophages, may contribute to a broad dysfunction of the circulating CD8^+^ T-cell pool that we and others have observed in chronic HCV infection [18,19,20,21]. While impaired HCV-specific CD8^+^ T-cells pose an impediment to virus control in chronic infection, a broad dysfunction of the T-cell compartment suggests an influence from antigen-independent cell-to-cell interactions or local and systemic microenvironments. We have recently reported that circulating CD8^+^ T-cells are hyperactivated in HCV^+^ individuals with advanced liver fibrosis, and that this is sustained long after HCV cure with IFN-free direct-acting antiviral therapy [21]. To date, it has been shown that macrophage subsets can polarize CD4^+^ T-cell responses to pro- or anti-inflammatory states [22,23,24,25,26], whereas their effects on CD8^+^ T-cells are less well-understood. Herein, we demonstrate impaired MDM subset differentiation in chronic HCV infection and our results suggest that macrophage dysfunction can contribute to observed CD8^+^ T-cell dysfunction.

## 2. Methods

### 2.1. Study Groups

Individuals recruited for the study were HCV-negative (HCV^−^) healthy volunteers and treatment-naïve HCV mono-infected individuals (detectable HCV RNA > 6 months, no HIV or hepatitis B virus (HBV) co-infection). HCV-infected individuals were classified based on the degree of liver fibrosis, determined diagnostically using the Metavir staging system with ultrasound elastography to ascribe an F-score (F0-F3-4) for HCV based on determined liver thickness (kPa). The HCV-infected individuals were grouped as follows: HCV^+^(F0-2) (F0-2, <9.0 kPa) or HCV^+^(F3-4) (F3-4, >11.0 kPa). The HCV^+^(F3-4) group was restricted to individuals with Child-Pugh class A liver disease. Peripheral blood was collected from study subjects attending the Viral Hepatitis Clinic and the Clinical Investigations Unit at The Ottawa Hospital. All subjects gave their informed consent for inclusion before they participated in the study. The study was conducted in accordance with the Declaration of Helsinki, and the protocol was approved by The Ottawa Health Science Network Research Ethics Board (protocol #20120009-01H, approved March 15, 2018) before undertaking this research. Patient characteristics are shown in Table 1.

### 2.2. Isolation of Monocytes and Generation of Macrophage Subsets

Subsets of MDM were generated as described previously [27,28]. Specifically, fresh peripheral blood mononuclear cells (3 × 10^6^ cells/mL) were cultured in serum-free Iscove’s modified Dulbecco’s Modified Eagles’ Medium (DMEM) (Thermo Fischer Scientific, New York, NY, USA) for 3 h to allow for monocyte adherence. Non-adherent cells were frozen for later use in CD8^+^ T-cell co-culture experiments. To the adherent monocytes, M-CSF was added (R&D Systems, Minnesota, MN, USA, 20 ng/mL) in complete DMEM (+10% FCS (fetal calf serum) + 10 units/mL of penicillin/streptomycin) for a 6-day culture to generate macrophages, replacing media after 4 days. On the seventh day, adherent macrophages were washed and incubated with Accutase cell detachment solution (Innovative Cell Technologies, California, CA, USA) for 30 min at 37 °C and gently removed with a cell scraper (Sarstedt Ag & Co, Numbrecht, Germany). Cells were cultured in complete DMEM 12-well polystyrene plates (Thermo Fischer Scientific, New York, NY, USA), (250,000 cells/mL) dispensing 1 mL cells/well. Polarizing cytokines were added to generate each of the four macrophage subsets (M1, M2a, M2b, and M2c) and the untreated cells were designated as M0. The following reagents were used to generate the macrophage subsets over a 48 h culture period: M1: IFN-γ, 20 ng/mL (Thermo Fischer Scientific, New York, NY, USA); M2a: IL-4, 20 ng/mL (R&D Systems, Minnesota, MN, USA); M2b: LPS, 1 μg/mL (Millipore Sigma, Oakville, ON, Canada) + IL-1β, 10 ng/mL (R&D Systems); M2c: IL-10, 10 ng/mL (R&D Systems).

### 2.3. Macrophage Subset Phenotyping by Flow Cytometry

To assess the cell surface phenotype of polarized macrophage subsets, cells were incubated with fluorescently-conjugated anti-CD163 (PE, clone GHI/61), anti-CD206 (APC, clone 15-2), and anti-CD86 (FITC, clone BU63) antibodies (BioLegend, California, CA, USA) for 15 min, in the dark, at 4 °C in phosphate-buffered saline (PBS) containing 1% bovine serum albumin (BSA) and 10% FCS, then fixed with 4% PFA (para-formaldehyde). Cell marker expression was evaluated using a FC500 NaviosTM flow cytometer (Beckman Coulter, Ontario, ON, Canada), applying Fluorescence Minus One controls, as appropriate for analysis.

### 2.4. Quantification of Secreted Cytokines

Cytokines were quantified using multiplex immunobead assay kits for analysis by the Magpix Luminex system (BioRad, Mississauga, ON, Canada) as per the manufacturer’s instructions. Interferon-γ and TNF-α were quantified with a screening assay kit (EMD Millipore, Etobicoke, Ontario, ON, Canada), while a high-sensitivity assay was used to quantify IL-10, IL-12, and IFN-γ (R&D Systems).

### 2.5. Macrophage:CD8^+^ T-Cell Co-Culture

After differentiation and polarization, MDM subsets (100,000 cells/mL, 500 μL/well) were co-cultured at a ratio of 1:4 with autologous CD8^+^ T-cells (2 × 10^6^ cells/mL) in 24-well polystyrene plates (Thermo Fischer Scientific, New York, NY, USA) with and without transwells separating MDM from CD8^+^ T-cells (Corning, New York, NY, USA). Blood CD8^+^ T-cells were isolated from frozen PBMC (EasySep™ Human CD8 Positive Selection Kit II, STEMCELL Technologies, Vancouver, British Columbia, BC, Canada). Cultures were maintained for 24–48 h. Distinct individuals were evaluated for each functional assay.

### 2.6. CD8^+^ T-Cell Functions

#### Quantification of IFN-γ^+^ and Perforin^+^ Cells and Detection of Degranulating (CD107a^+^) Cells

The intracellular expression of IFN-γ in CD8^+^ T-cells was evaluated after 24 h of macrophage:CD8^+^ T-cell co-culture by flow cytometry. Six hours prior to the end of the co-culture, cells were treated with Brefeldin A (15 μg/mL, Millipore SIGMA, Oakville, ON, Canada) to prevent the release of IFN-γ or labeled with anti-human CD107a-FITC antibody or IgG1κ-FITC (clones H4A3 and MOPC-21, respectively, BD Biosciences, San Jose, CA, USA) while being treated with monensin (8 μM, BD Bioscience) to allow for the accumulation of CD107a on the cell surface if granule exocytosis were to occur. After labeling cells with anti-CD8 antibodies, cells were permeabilized with saponin (Millipore SIGMA) in 10% Human AB serum (Valley Biomedical Inc., Winchester, VA, USA) + anti-human IFN-γ-FITC antibodies (clone 4S.B3, BioLegend) or an IgG1κ-FITC isotype control (clone MOPC-21, BioLegend). To quantify perforin^+^ cells after 48 h of culture, cells were labeled with anti-human perforin-FITC antibodies (clone δG9, BD Pharmingen, BD Bioscience, San Jose, CA, USA) or IgG2b- FITC isotype control antibodies (clone 27-35, BD Pharmingen, BD Bioscience) followed by anti-CD8 antibody labeling. To minimize nonspecific binding, human AB serum (10%) was included in all buffers. Samples were analyzed by flow cytometry and data were analyzed using FCS Express Research Edition 4.0 (De Novo Software, Los Angeles, California, CA, USA). The release of perforin and IFN-γ into culture supernatants was also determined by an immunobead assay (Magpix) as above.

### 2.7. Statistical Analysis

Standard curves and protein quantification were calculated through an automatic report generation using the xPONENT^®^ on the MagpixTM system. Graphs were generated, and statistical tests performed, using the GraphPad Prism 5.0 software (San Diego, California, CA, USA). For all statistical tests, a paired or unpaired Student’s *t*-test was used as appropriate (*p* ≤ 0.05) unless otherwise specified. Where necessary, Multivariate Data Analysis and a one-way ANOVA Dunnett post-test were carried out. Data are presented as mean ± SD.

## 3. Results

### 3.1. Altered Phenotypic Surface Marker Expression on Macrophage Subsets from Chronic HCV-Infected Patients

**Figure 1:**
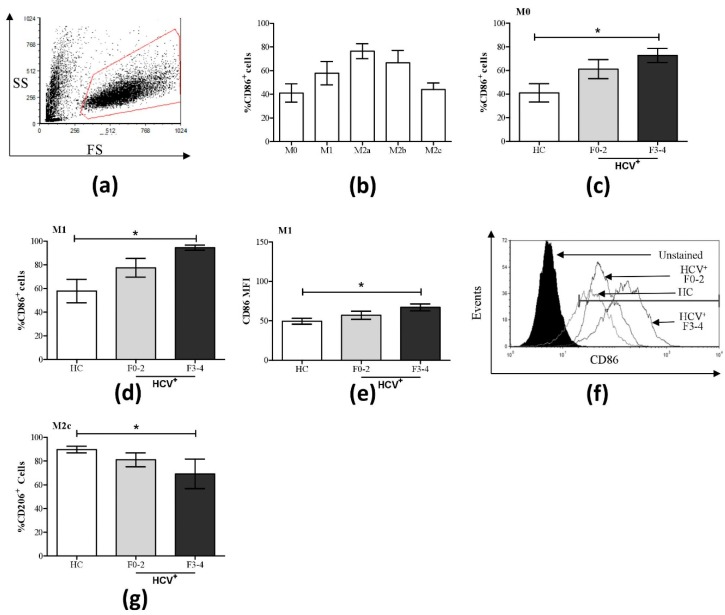
Increased percentage of CD86^+^ cells in M0 and M1 macrophage subsets and decreased CD206 expression in M2c cells in HCV infection. The expression of CD86 and CD206 was assessed on macrophage subsets from healthy controls (HC, n = 9) and HCV-infected individuals with minimal (F0-2, n = 9) or advanced liver fibrosis (F3-4, n = 4) by flow cytometry. (**a**) A representative dot plot of macrophage flow cytometry gating based on forward and side scatter is shown. (**b**) The proportion (%) of CD86^+^ cells across all macrophage subsets from healthy individuals is shown. Significant changes in % CD86^+^ cells in HCV^+^ study groups are shown for (**c**) M0 and (**d**) M1 cells. (**e**) Included is the degree of CD86 expression (mean fluorescence intensity, MFI) in M1 cells, which is accompanied by (**f**) a representative histogram with overlapping data traces from an uninfected donor and HCV-infected individuals with minimal or advanced liver fibrosis. (**g**) Significant changes in the % CD206^+^ cells were also found in the M2c subset. Statistical significance was determined in healthy controls by one-way, paired Student’s *t*-tests, and significance among HCV-infected groups was determined by a one-way ANOVA (*p* ≤ 0.05). Significant *p*-values are indicated with an asterisk “*”.

We have previously shown that this culture system polarizes human macrophages into various subsets, on the basis of an extensive assessment of cell surface receptors (CD14, CD80, CD86, CD163, CD200, and TLR4) and cytokine expression (IFN-γ, IL-1β, IL-2, -4, -5, -6, -9, -10, -12p70, -13, -17a, -22, -23, and TNF-α) [29]. In the present experiments, evidence of polarization could be readily seen in the morphological changes of the cultures, with polarized subsets taking on the characteristic spindle nature compared to the rounded features of unpolarized macrophages (Figure S1). Following a 6-day MDM differentiation and a 48-h polarization protocol, the expression of the surface receptor markers CD86, CD206, and CD163 of putative macrophage subsets was assessed. In controls, all macrophage subsets expressed CD86, with M2a and M2b cells expressing the highest proportion (%) of CD86^+^ cells compared to nonpolarized M0 cells (Figure 1a,b, Figures S2 and S3). The expression of CD86 alone does not distinguish macrophage subsets. The expression of the mannose receptor CD206 was relatively similar across MDM subsets in controls, ranging from approximately 75–90% expression levels (Figure 1g and Figures S4 and S5). There was a hierarchy of expression for the scavenger receptor CD163 across MDM subsets in controls (M2c > M2b > M0 and M1 > M2a, Figures S6 and S7).

A statistical trend suggested an increase of CD86^+^ M1 cells in HCV^+^(F0-2) individuals compared to controls (*p* = 0.08), and statistically significant increases in CD86^+^ M0 and M1 cells in HCV^+^(F3-4) individuals (*p* = 0.03 and 0.02, respectively, Figure 1c,d) were observed; levels that are more comparable to that of the M2a subset in controls. Increased CD86 expression in HCV^+^(F3-4) individuals was also observed on a per-cell basis in M1 cultures, as measured by mean fluorescence intensity (MFI, *p* = 0.01, Figure 1e). The expression of CD86 in M2a, M2b, and M2c was not significantly altered between controls and HCV^+^ groups (Figures S2 and S3).

The proportion of cells expressing CD206 was significantly reduced in M2c macrophages from HCV^+^(F3-4) individuals, while other MDM subsets showed no significant differences compared to controls (Figure 1g, Figures S4 and S5). Individual variation in CD163 expression levels precluded the statistical significance of possible increases in CD163^+^ M0 and M1 cells in HCV^+^ individuals to levels similar to that of M2c cells in controls (Figures S6 and S7).

### 3.2. Altered Pro- and Anti-Inflammatory Cytokine Expression on Macrophage Subsets from HCV-Infected Patients

**Figure 2:**
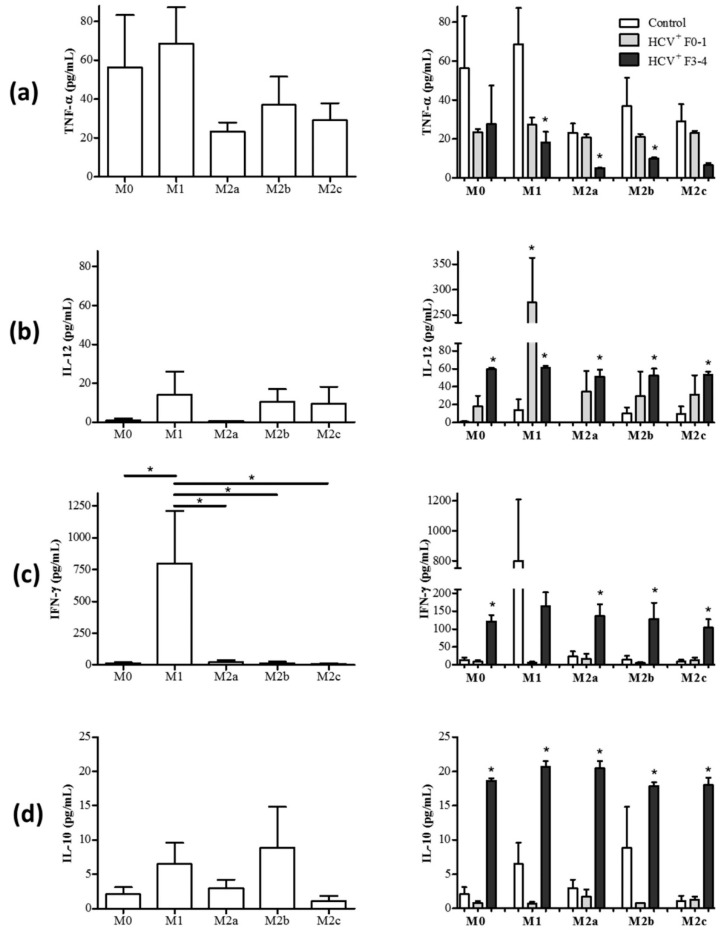
M1 macrophages in HCV-infected individuals acquire M2-like cytokine secretion features while M2 macrophages acquire M1-like cytokine features. Macrophage subsets from healthy controls (HC, n = 6–7) and chronic HCV-infected individuals with minimal liver fibrosis (n = 4) or advanced liver fibrosis (n = 3–4) were cultured for 72 h, after which culture supernatants were collected. The concentration of (**a**) TNF-α, (**b**) IL-12, (**c**) IFN-γ, and (**d**) IL-10 in culture supernatants were quantified using multiplexing immunobead assays. Panels on the left display data relating to the secretion of these cytokines by each macrophage subset as follows: M0, M1, M2a, M2b, and M2c. Panels on the right show data from monocyte-derived macrophage (MDM) cultures derived from HCV^+^ individuals. Statistical significance of cytokine secretion by cells from controls was determined by one-tailed paired Student’s *t*-test, HCV^+^ groups were compared to uninfected controls using a one-way ANOVA (*p* ≤ 0.05), and significant *p*-values are indicated with an asterisk “*”. Any significant differences between HCV^+^ study groups are also indicated with a line and an asterisk.

While the expression of cell surface receptors can be somewhat variable in determining macrophage subset polarization in humans, the expression of cytokines can confer a better appreciation of functional polarization. To further determine if MDM subsets are further phenotypically and functionally altered in HCV infection, the cytokine production profiles of MDM subset cultures were determined. After the 48 h polarization of MDM, the polarizing stimuli were removed by aspirating culture supernatants and fresh medium was added for a further 24 h incubation, as described previously [29]. The concentration of TNF-α, IL-12, IFN-γ, and IL-10 in culture supernatants were quantified by immunobead assay. The secretion profiles of TNF-α, IL-12, IFN-γ, and IL-10 in MDM subsets derived from healthy controls are shown in Figure 2a–d. M1 MDM secreted a high concentration of IFN-γ, whereas M2 subsets expressed very low concentrations of IFN-γ (Figure 2c). The concentration of IL-12p70 was detectable in M1, M2b, and M2c cells (Figure 2b). There were no significant differences detected in the release of TNF-α across the macrophage subsets (Figure 2a), and none of the subsets produced significantly high levels of IL-10 (Figure 2d).

#### 3.2.1. M1 Cells Acquire Cytokine Secretion Features of M2 Cells in HCV^+^(F3-4) Individuals

An examination of MDM cultures derived from HCV^+^ individuals and polarized with M1 stimuli revealed changes in cytokine secretion to resemble that of M2 MDM. There was a significant reduction in the release of TNF-α in M1 cultures compared to controls (average 68.6 pg/mL), particularly in the HCV^+^(F3-4) study group (average 18.3 pg/mL, *p* = 0.04), and a trend suggested a decrease in the HCV^+^(F0-2) study group (average 27.3 pg/mL, *p* = 0.07, Figure 2a, right panel). The production of pro-inflammatory IFN-γ by putative M1 cells derived from HCV-infected individuals seemed to be reduced (HCV^+^ (F3-4) average 164.0 pg/mL) compared to controls (average 798.5 pg/mL), although this did not reach statistical significance (Figure 2c, right panel). The production of anti-inflammatory IL-10 was also increased significantly in M1 cultures from HCV-infected individuals compared to controls (Control: average 6.5 pg/mL versus HCV^+^ (F3-4): average 20.7 pg/mL, p = 0.006, Figure 2d). Meanwhile, the M1 cells derived from HCV-infected individuals maintained an M1-like production of IL-12, secreting significantly higher concentrations than controls (Control: average 14.2 pg/mL versus HCV^+^ (F0-2): 274.8 pg/mL and (F3-4) 61.1 pg/mL, *p* = 0.01 in each HCV^+^ group, Figure 2b). In healthy controls, the M0 subset produced very low concentrations of IL-12p70, IFN-γ, and IL-10 (Figure 2b–d), yet in cells from HCV-infected individuals these levels increased significantly in the (F4) group (Figure 2b–d, right panel). The secretion of TNF-α by M0 cells in healthy individuals was comparable to that of M1 cells (Figure 2a) and was not significantly altered in HCV infection.

#### 3.2.2. M2 Cells Acquire Cytokine Secretion Features of M1 Cells in HCV^+^(F3-4) Individuals

The polarization of MDM cells from HCV-infected individuals with M2 stimuli resulted in the acquisition of certain M1-like cytokine secretion profiles. The release of IL-12 was significantly increased in all M2 subsets, in a statistically significant manner compared to controls (HCV^+^ (F3-4) M2a *p* < 0.0001, M2b *p* = 0.003, and M2c *p* = 0.004, Figure 2b). In addition, all M2 subsets derived from HCV^+^ individuals significantly increased the release of IFN-γ (up to 230 pg/mL) compared to controls (average <25 pg/mL) (HCV^+^ (F3-4) *p* < 0.0001, Figure 2c). The M2 subsets in HCV infection continued to release anti-inflammatory IL-10 at concentrations that were significantly greater than controls (*p* < 0.0001 for HCV^+^ (F3-4), Figure 2d). In the case of M2b, cells from HCV^+^ (F3-4) individuals secreted more IL-10 than HCV^+^ (F0-2) individuals, which were no different than controls (*p* < 0.0001). The M2a and M2b subsets also maintained a comparatively low level of TNF-α secretion in HCV^+^(F3-4) (i.e., compared to controls, *p* = 0.03 and 0.01, respectively) and a lower level of TNF-α secretion than M1 cells in controls or HCV infection, whereas any change in concentrations secreted by M2c cells did not reach statistical significance (Figure 2a, right panel).

### 3.3. M1 Macrophages Enhance CD8^+^ T-Cell Functions in Normal Healthy Individuals via Contact-Dependent Mechanisms

**Figure 3:**
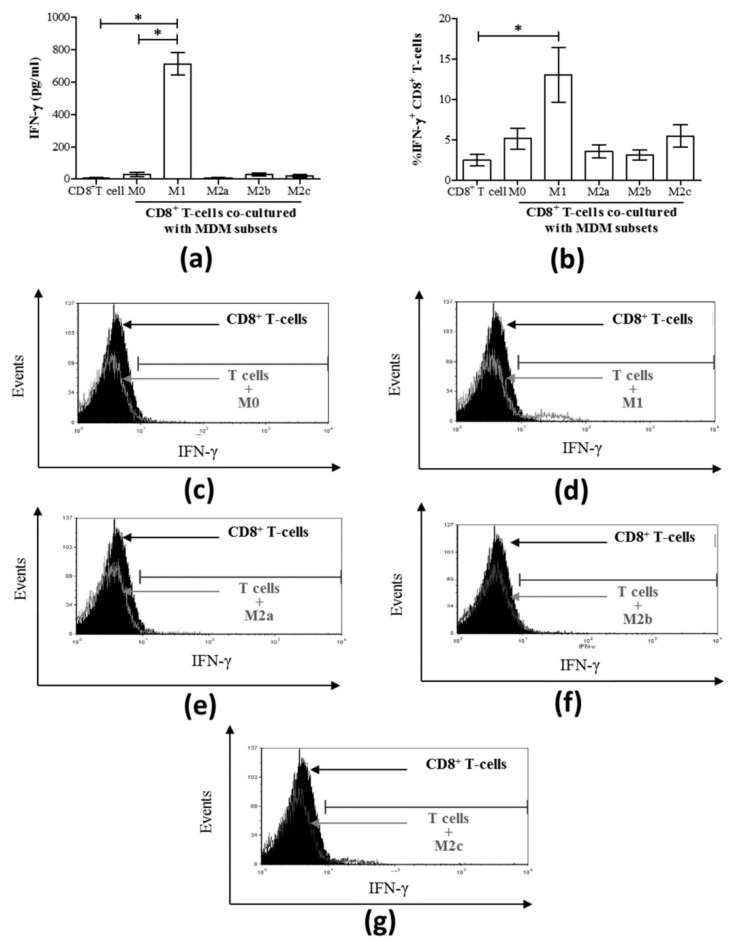
The release of IFN-γ and the proportion (%) of IFN-γ-expressing CD8^+^ T-cells are increased when co-cultured directly with M1 macrophages derived from uninfected individuals. MDM subsets (50 × 10^3^) were directly co-cultured with autologous CD8^+^ T-cells (200 × 10^3^) for 24 h (n = 3). (**a**) The concentration of IFN-γ released into culture supernatants was quantified by an immunobead assay and the results are graphed, including T-cells cultured alone. (**b**) The proportion of IFN-γ^+^ CD8^+^ T-cells after 24 h of macrophage:T-cell co-culture was determined by intracellular staining and flow cytometry, and data are summarized in a graph. Statistical significance was determined by a Student’s *t*-test (*p* ≤ 0.05), and significant responses are indicated with a line and asterisk “*”. Representative histograms of intracellular IFN-γ expression by CD8^+^ T-cells co-cultured with the following macrophage subsets: (**c**) M0, (**d**) M1, (**e**) M2a, (**f**) M2b, and (**g**) M2c, n = 7. These histograms include traces for CD8^+^ T-cells cultured alone (filled black area).

IFN-γ production is a central function of CD8^+^ T-cells and an important modulator of anti-viral responses [30]. To evaluate the impact of macrophages and their subsets on the release of IFN-γ in co-culture with CD8^+^ T-cells, polarized macrophages were washed and co-cultured in fresh culture medium with autologous CD8^+^ T-cells for 24 h, after which IFN-γ production was quantified in the culture supernatants. While T-cells alone did not spontaneously release IFN-γ once cultured with M1 subsets, the two cell types together consistently produced high concentrations of IFN-γ (mean 711.8 pg/mL ± 68.38 standard error (S.E.), Figure 3a), which was significantly higher than M0 cells or CD8^+^ T-cells alone (*p* = 0.01). Since M1 macrophages alone produced significantly high levels of IFN-γ (Figure 2c), it was not clear whether the enhanced IFN-γ production detected in the M1-CD8^+^ T-cell co-culture experiments was indeed derived from the CD8^+^ T-cells. To demonstrate that CD8^+^ T-cells were a source of the detected IFN-γ in these co-cultures, the proportion of IFN-γ^+^ CD8^+^ T-cells was determined by flow cytometry analysis by intracellular staining (Figure 3b). The contribution of CD8^+^ T-cells to the IFN-γ detected in M1 co-cultures was evident by a significant increase in the proportion of IFN-γ CD8^+^ T-cells compared to CD8^+^ T-cells alone (*p* = 0.01). Co-culture with M2 MDM subsets did not result in the release of IFN-γ. Representative histograms of IFN-γ-expressing CD8^+^ T-cells following co-culture with each MDM subset are shown in Figure 3c–g.

**Figure 4:**
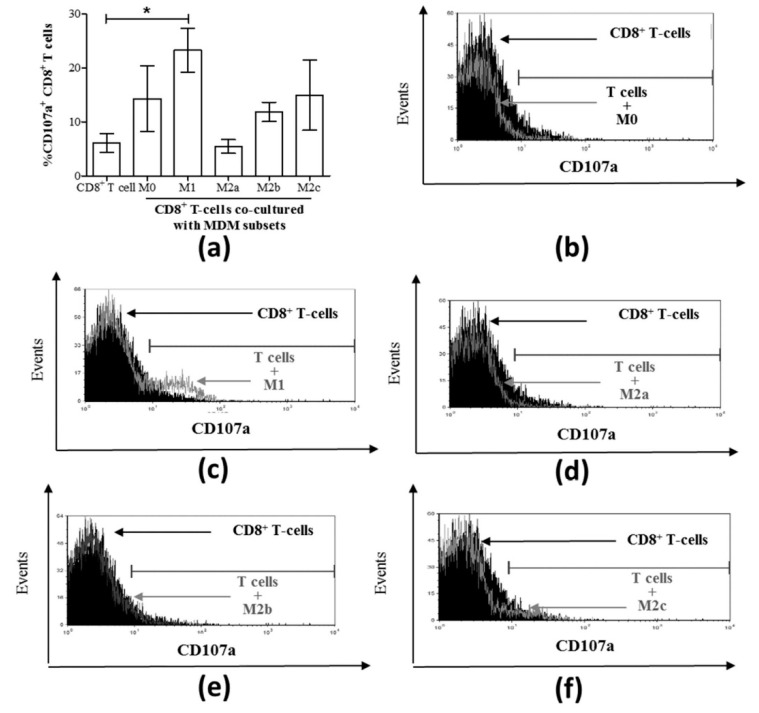
Only M1 macrophages cultured with CD8^+^ T-cells increased the proportion of CD107a^+^ CD8^+^ T-cells. (**a**) MDM subsets were directly cultured with isolated autologous CD8^+^ T-cells for 48 h, and the expression of surface CD107a on CD8^+^ T-cells was assessed by flow cytometry (n = 6). Data are summarized in a graph depicting the proportion of cells expressing CD107a. Representative histograms are shown for each co-culture of T-cells with the following macrophage subsets: (**b**) M0, (**c**) M1, (**d**) M2a, (**d**) M2b, and (**f**) M2c. The histograms include traces for CD8^+^ T-cells cultured alone (filled black area) superimposed by co-culture traces (grey outlines). Statistical significance was determined by a Student’s *t*-test (*p* ≤ 0.05), and significant responses are indicated with a line and an asterisk “*”.

We then evaluated CD8^+^ T-cell degranulation potential in MDM subset co-cultures by measuring CD107a expression (Figure 4a). A significant increase in the proportion of CD107a^+^ CD8^+^ T-cells was detected in M1 macrophage co-cultures compared to CD8^+^ T-cells cultured alone as controls (*p* = 0.003, Figure 4a,c). None of the other MDM subset co-cultures resulted in significant degranulation of CD8^+^ T-cells compared to the control cells (Figure 4a). Representative histograms of CD107a-expressing CD8^+^ T-cells following co-culture with each MDM subset are shown in Figure 4b–f.

**Figure 5:**
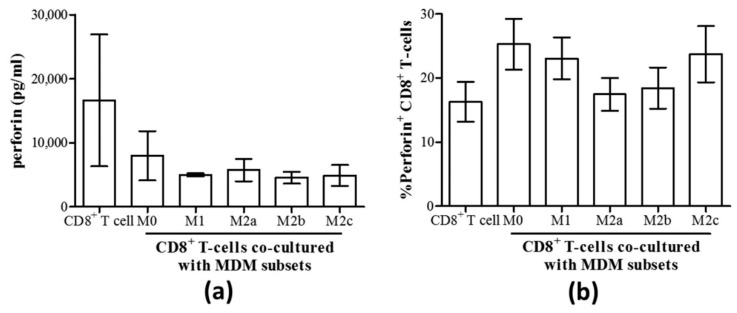
Perforin production by CD8^+^ T-cells remained unaffected by the co-culture with any macrophage subset. (**a**) The concentration of perforin released into culture supernatants of isolated autologous CD8^+^ T-cells cultured with MDM subsets for 48 h was quantified by an immunobead assay (n = 3). (**b**) The proportion of perforin^+^ CD8^+^ T-cells in co-cultures with macrophage subsets is shown (n = 7).

As degranulation can involve the release of the lytic protein perforin, the production of perforin by CD8^+^ T-cells and its release were evaluated in MDM co-cultures. We found no differences in the concentration of perforin released into culture supernatants by CD8^+^ T-cells co-cultured with any MDM subset compared to CD8^+^ T-cells alone (Figure 5a). Similarly, there were no differences in the proportion of perforin^+^ CD8^+^ T-cells in these co-cultures, as determined by intracellular expression of perforin (Figure 5b).

**Figure 6:**
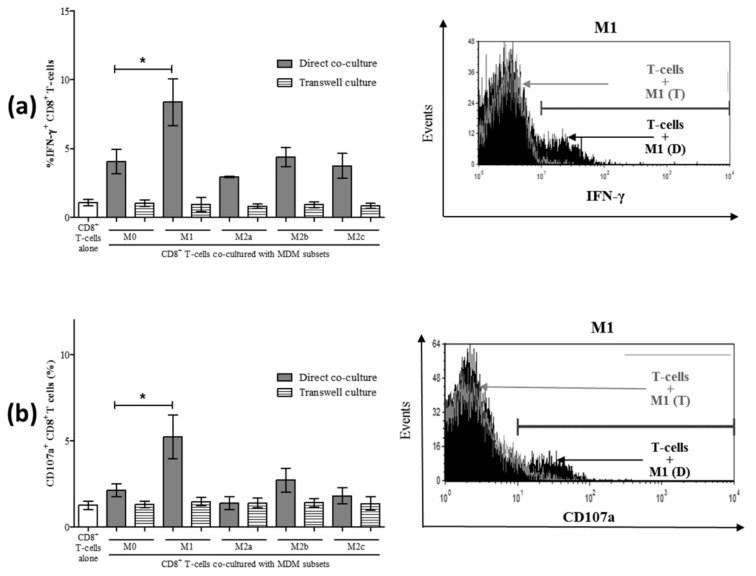
Increased IFN-γ and CD107a expression by CD8^+^ T-cells following co-culture with macrophage subsets is contact-dependent. Macrophage subsets were co-cultured either directly with autologous CD8^+^ T-cells or were separated by a transmembrane. After 24 h of culture, intracellular expression of IFN-γ was evaluated in CD8^+^ T-cells by flow cytometry. (**a**) The proportion (%) of IFN-γ^+^ CD8^+^ T-cells following either direct co-culture (grey bars) or co-cultures separating MDM from CD8^+^ T-cells in a transwell (hatched bars) is shown in the summary graph (n = 3). A representative histogram depicts intracellular IFN-γ expression by CD8^+^ T-cells in direct and transwell co-culture with M1 macrophages and includes a trace for CD8^+^ T-cells cultured alone (filled black area). (**b**) After 48 h of direct or indirect co-culture of macrophage subsets with CD8^+^ T-cells, the expression of CD107a was evaluated by flow cytometry. A representative histogram compares the CD107a expression of CD8^+^ T-cells in direct and transwell co-culture with M1 macrophages and includes a trace for CD8^+^ T-cells cultured alone (filled black area). Statistical significance was determined by a Student’s *t*-test (*p* ≤ 0.05), and significant *p*-values are indicated with an asterisk “*”.

To determine the mechanism by which normal M1 macrophages enhance CD8^+^ T-cell function (i.e., enhanced IFN-γ production and CD107a expression), we investigated whether this occurred through soluble means or cell-to-cell contact. Direct co-culture of MDM subsets with CD8^+^ T-cells, as described above, was conducted in parallel with co-cultures in transwell culture plates separating MDM cells from CD8^+^ T-cells by a 0.4 μm transmembrane to permit the diffusion of soluble molecular messengers. Compared to CD8^+^ T-cells alone, co-culture with unpolarized M0 MDM increased the proportion of IFN-γ^+^ CD8^+^ T-cells (*p* < 0.02). In terms of the effect of MDM polarization on CD8^+^ T-cell activity, a significant increase in the proportion of IFN-γ^+^ CD8^+^ T-cells was detected in direct co-cultures with M1 macrophages (Figure 6a, *p* = 0.04) compared to M0 co-cultures and consistent with our previous observations (Figure 3a,d). The M2 subsets did not change the proportion of IFN-γ^+^ CD8^+^ T-cells compared to M0 co-cultures. The effect was lost in transwell co-cultures, wherein there was no significant change in the proportion of IFN-γ^+^ CD8^+^ T-cells cultured with MDM compared to CD8^+^ T-cells alone. When assessing degranulation in these same cultures, we also observed a contact-dependent induction of CD107a^+^ CD8^+^ T-cells by M1 macrophages, and this effect was lost in the transmembrane co-culture system (Figure 6b). None of the other macrophage subsets significantly induced CD8^+^ T-cell degranulation in either direct or transmembrane co-cultures. These results suggest that the mechanism by which M1 macrophages enhance spontaneous CD8^+^ T-cell functions, such as IFN-γ and CD107a induction, is contact-dependent.

### 3.4. M1 Macrophages Inhibit IFN-γ Expression on CD8^+^ T-Cells in Chronic HCV Infection

**Figure 7:**
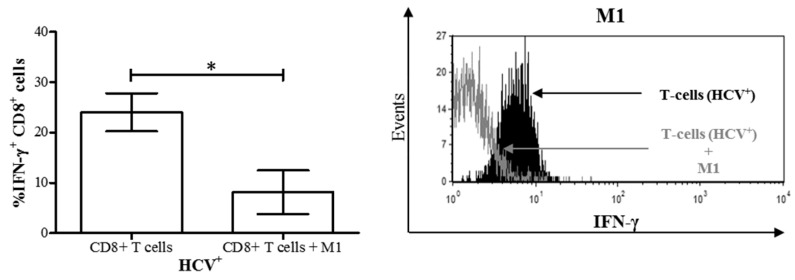
M1 macrophages derived from HCV-infected individuals with minimal liver fibrosis decrease the proportion of IFN-γ^+^ CD8^+^ T-cells in co-culture. After differentiation from MDMs derived from blood monocytes from HCV-infected individuals, M1 macrophages were co-cultured for 24 h with autologous CD8^+^ T-cells isolated from frozen peripheral blood mononuclear cells. Intracellular IFN-γ expression in CD8^+^ T-cells was then evaluated by flow cytometry. The proportion of IFN-γ^+^ CD8^+^ T-cells in co-cultures was compared to that of CD8^+^ T-cells cultured alone (n = 7). Representative histograms of IFN-γ expression by CD8^+^ T-cells co-cultured with M1 macrophages (unfilled area) superimposed over CD8^+^ T-cells cultured alone (black filled area) are also shown. Statistical significance was determined by a Student’s *t*-test (*p* ≤ 0.05), and statistical significance is indicated with an asterisk “*”.

We have previously reported impairment of bulk CD8^+^ T-cell cytokine signaling, survival, and more recently function in chronic HCV infection [20,21]. We then endeavored to determine if the altered differentiation of MDM polarized with M1 stimuli in chronic HCV infection influenced CD8^+^ T-cell function. MDM M1 cells derived from treatment-naïve HCV^+^ individuals were co-cultured with autologous CD8^+^ T-cells as above. After 48 h of culture, we observed a significant decrease in IFN-γ^+^ CD8^+^ T-cells in M1 co-cultures compared to autologous CD8^+^ T-cells cultured alone (Figure 7). Furthermore, the proportion of IFN-γ^+^ cells in these co-cultures was significantly lower than that of controls (Figure 3b, *p* < 0.05). This suggests that the dysregulated differentiation of M1 cells in HCV infection inhibited IFN-γ expression on CD8^+^ T-cells from HCV-infected individuals.

## 4. Discussion

This report demonstrates a significant role of M1 macrophages in mediating cytolytic and immunomodulatory functions (IFN-γ expression and cell degranulation, CD107a) of human CD8^+^ T-cells from healthy individuals and highlights their contribution to CD8^+^ T-cell dysfunction in chronic HCV infection. We also show several phenotypic alterations in M1 differentiation from MDM in HCV infection that were associated with the severity of liver disease. In HCV^+^(F3-4), M1 macrophages exhibited an M2-like anti-inflammatory state as evidenced by decreased pro-inflammatory characteristics, such as IFN-γ, TNF-α, and IL-12 production, an increase in anti-inflammatory IL-10 production, and an increased proportion of the co-stimulatory receptor CD86. In contrast, M2a, 2b, and 2c macrophage subsets exhibited increased IFN-γ and IL-12 production. Finally, while M1 macrophages enhanced IFN-γ expression by CD8^+^ T-cells in health; an inhibition of this important cell function was observed with cells from HCV-infected individuals. This identifies a cell-to-cell interaction that underlies the observed impairment of CD8^+^ T-cells in chronic HCV infection, and that may be associated with the severity of liver disease.

Several changes to the phenotype and cytokine profiles of MDM-derived subsets were observed when comparing cells from controls and HCV-infected patients, finding most HCV-related changes in cells derived from HCV^+^(F3-4) individuals. The proportion of CD86^+^ cells and CD86 expression levels were significantly higher in M0 and M1 macrophages (Figure 1c–f) in HCV^+^(F3-4) compared to controls. This suggests that an altered M1 phenotype in chronic HCV infection is associated with the severity of liver disease. The impact of increased CD86 expression on M1 macrophages on the immune system in chronic HCV infection is poorly understood. Increased CD86 expression has been observed in a somewhat similar chronic, hepatotropic infection with HBV, where liver lobules were found to have a higher total count and percentage of CD86^+^ macrophages, compared to CD80^+^ or PD-L1^+^ macrophages [31]. In murine models, while CD80 is known to drive a Type 1 (Th1) cytokine response from T-cells, CD86 is expected to drive a Type 2 (Th2) response in CD4^+^ T-cells [32]. In fact, CD86 was unable to activate CD8^+^ T-cells effectively [33].

Macrophages are one of the main producers of the pro-inflammatory cytokine TNF-α. Moreover, M1 cells spontaneously secrete TNF-α [34]. In tumor microenvironments, changes in TNF-α mRNA expression and Type I TNF receptor signaling modulate MDM differentiation to favor the M2 phenotype [35]. In our studies, M1 macrophages from HCV^+^(F3-4) individuals spontaneously produced less TNF-α than controls (Figure 2). Additionally, although macrophages in general do not produce the pro-inflammatory cytokine IFN-γ, M1 macrophages from healthy individuals secreted significantly higher concentrations of IFN-γ (Figure 2c). However, IFN-γ production was significantly reduced after M1 polarization in HCV infection. The reduced production of IFN-γ and TNF-α suggests the loss of defining pro-inflammatory traits of M1 macrophages in HCV infection. Interestingly, the increased production of IFN-γ by M0 and M2 macrophage subsets and IL-12p70 sheds light on the altered functionality of M2 macrophages towards a pro-inflammatory state in chronic HCV infection. This is also supported by M2c macrophages reducing CD206 expression, also a feature of M1 subsets [36]. Increased expression of IFN-γ mRNA in livers of chronic HCV patients has been observed, particularly in areas with inflammation and fibrosis [37]. The increased production of anti-inflammatory IL-10 in both M1 and M2 subsets further supported our observations in HCV infection that M1-stimuli yielded MDM cells with some M2 characteristics (e.g., increased CD86 expression, less TNF-α and IFN-γ, increased IL-10), while M2 subsets exhibited more M1 characteristics (e.g., reduced CD206 expression, increased IFN-γ and IL-12), particularly in cells from HCV^+^(F3-4) individuals. Previous studies have shown decreased IL-12 production by macrophages in HCV infection, although these did not differentiate between specific MDM subsets [38,39]. The release of IL-12 was increased in M1 MDM cultures from both HCV groups, yet increases in IL-10 were restricted to the HCV^+^(F3-4) group (Figure 2).

The possible mechanism underlying the observed impairment remains unknown. In vitro studies have reported that certain viral proteins (e.g., HCV core and NS3) trigger increased production of TNF-α, IL-12, and IL-10 by blood monocytes. HCV core protein has been implicated in preventing complete differentiation of monocytes to M1 or M2 macrophages by modulating STAT signaling pathways [39,40]. In addition, molecular factors secreted by HCV-infected cells have been shown to mediate M2-polarization [12], suggesting that soluble factors could influence cell function. The involvement of HCV-specific factors affecting MDM differentiation in chronic HCV infection needs to be further investigated.

It is well-established that macrophages activate T-cells by presenting antigen peptides in the context of MHC molecules, and T-cell activation is aided in part by the cytokine microenvironment [41]. Furthermore, macrophages can activate CD8^+^ T-cells independently of antigen recognition [42]. Our results suggested, for the first time, that the functions of CD8^+^ T-cells (i.e., IFN-γ and CD107a expression) can be induced upon culture with M1 macrophages in a cytokine- and antigen-independent manner in health. It was surprising that changes in perforin expression by CD8^+^ T-cells were not observed alongside the detectable degranulation marker CD107a. This may be attributed to either the timing of the assay or different combinations of granzymes with and without perforin or the need for a second signal to enable cytolytic molecule release [43]. Antigen-independent induction of CD8^+^ T-cell proliferation in co-cultures with IL-15-producing dendritic cells presenting self-peptides in murine models of lymphopenia has been demonstrated [44]. Whether similar presentation of self-peptides by M1 macrophages is involved in their induction of IFN-γ and CD107a expression by CD8^+^ T-cells here remains unclear.

The mechanism by which M1 macrophages reduced the proportion of IFN-γ^+^ CD8^+^ T-cells following co-culture in chronic HCV infection while inducing IFN-γ expression in controls is not clear. Murine M1-like KCs impair tumor-specific CD8^+^ T-cell IFN-γ production [45,46], suggesting that chronic disease alters MDM subset functions. Our data, and those of others, suggest that altered M1 macrophage differentiation results in the inhibition of adaptive immune responses [47]. For instance, increased expression of inhibitory ligands (such as PD-L1) on M1 macrophages [31] could result in contact-mediated exhaustion of the IFN-γ^+^ CD8^+^ T-cells. In turn, CD8^+^ T-cells could certainly influence the polarization of macrophage subsets (not evaluated here), as macrophage subset reversal can occur readily due to the inherent plasticity of these cells [48]. The increased IFN-γ produced by CD8^+^ T-cells in HCV infection in these experiments could polarize M2 subsets towards an M1 phenotype secreting IFN-γ. Interferon-γ is also known to induce immunosuppression in macrophages [49], which may have resulted in the downregulation of IFN-γ^+^ T-cells in the co-culture. Therefore, dysfunctional macrophages could influence CD8^+^ T-cell function, as shown here, while it is also possible that dysfunctional CD8^+^ T-cells may affect macrophage polarization, but this has not been documented. However, it has been shown that monocytes from HCV-infected individuals can repolarize macrophages to the M2 phenotype, resulting in decreased IFN-γ production [50]. This would concur with our interpretation that macrophages from HCV-infected individuals that were stimulated with M1-polarizing agents acquired M2 characteristics and inhibited IFN-γ production by CD8^+^ T-cells in co-culture (Figure 7). Furthermore, we have previously shown that, in HCV infection, CD8^+^ T-cells are vulnerable to apoptosis [20] and apoptotic CD8^+^ T-cells have been shown to shift macrophages towards the M2 phenotype [51]. Finally, whether macrophages are phagocytosing apoptotic cells, as has been described [52], was not evaluated here. We have previously observed that CD8^+^ T-cells of HCV-infected individuals express less Bcl-2 than healthy controls, and hence are certainly likely to be more susceptible to apoptotic signals [53]. Whether this occurred in the short period of co-culture used here was not determined.

In summary, this report demonstrates the reprogramming of macrophage subsets derived from HCV-infected individuals. The M1 subset from normal healthy individuals can enhance CD8^+^ T-cell functions, in a cytokine- and antigen-independent, contact-mediated manner, yet this effect is abrogated in chronic HCV infection. The molecular mechanism underlying various phenotypic alterations identified among MDM subsets in chronic HCV infection remain to be investigated. This should include an assessment of potential effects of HCV infection of monocytes on subset differentiation and function [54,55]. The relevance of liver disease severity in altered macrophage differentiation is also an intriguing observation. Collectively, this research will lead to identifying possible therapeutic targets to restore immune function in HCV-infected individuals, particularly to circumvent the lack of a potent IFN-γ response in the liver. Assessment of innate lymphoid cells may also be of value given their recent implication in mediating liver immune tolerance in HCV infection [56] and impairment of potent CD8^+^ T cell responses to viral hepatitis in a murine model [57].

## Figures and Tables

**Table 1:** Characteristics of healthy controls and hepatitis C virus (HCV)-infected individuals.

	Healthy Controls	HCV^+^ (F0-2)^1^	HCV^+^ (F3-4)^2^
**Number**	13	9	4
**Age, avg. years (SD^3^)**	31.2 (8.4)	50 (14.9)	56.5 (4.1)
**Race**	Caucasian	Caucasian (7), East Indian (1), African (1)	Caucasian (3), Native (1)
**HCV-RNA (IU/mL)**		3.1 × 10^6^ (2.2 × 10^6^)	1.6 × 10^6^ (2.2 × 10^6^)
**HCV genotype^4^ (n)**		1 (7: 1a n = 3, 1b n = 1), 2b (1)	1a (3), 3 (1)
**Fibroscan, avg. kPa^5^ (SD)**		6.1 (1.9)	19.3 (7.1)
**AST^6^ (U/L)**		25.4 (12.9)	47.3 (37.3)
**ALT^7^ (U/L)**		47.3 (23.6)	75.0 (42.0)

^1^ (F0-2): minimal liver fibrosis (<9.0 kPa); ^2^ (F3-4) advanced liver fibrosis (>11.0 kPa); ^3^ SD (standard deviation); ^4^ not all HCV subgenotypes were known; ^5^ kPa (kilopascal); ^6^ AST (Aspartate aminotransferase); ^7^ ALT (Alanine aminotransferase).

## Supplementary Materials

The following are available online at https://www.mdpi.com/2073-4409/8/4/374/s1, Figure S1: Macrophage morphology in vitro; Figure S2 Expression of CD86 in MDM subsets from uninfected controls and HCV-infected individuals; Figure S3 Mean fluorescence intensity of CD86 expression on macrophage subsets from uninfected controls and HCV-infected individuals; Figure S4 Expression of CD206 on macrophage subsets from uninfected controls and HCV-infected individuals; Figure S5 Mean fluorescence intensity of CD206 expression on macrophage subsets from uninfected controls and HCV-infected individuals; Figure S6 Expression of CD163 on macrophage subsets from uninfected controls and HCV-infected individuals; Figure S7 Mean fluorescence intensity of CD163 expression on macrophage subsets from uninfected controls and HCV-infected individuals.
