# Supplementary material for: Chronic Hepatitis C Virus Infection Impairs M1 Macrophage Differentiation and Contributes to CD8+ T-Cell Dysfunction
Source: Cells. 2019 Apr 25;8(4):374. doi: 10.3390/cells8040374 (PMC6523920; doi:10.3390/cells8040374)
Supplement: Supplementary file 1 [file cells-08-00374-s001.pdf]

Supplemental figures:

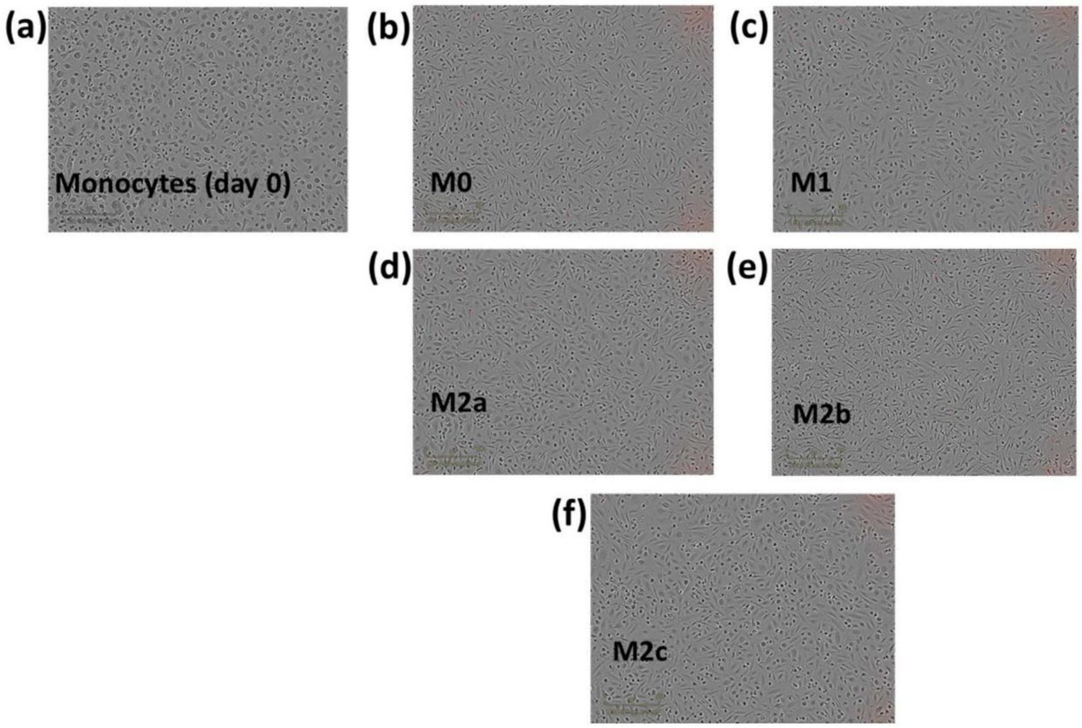

Figure S1. Macrophage morphology in vitro. The morphology of (**a**) adherent monocytes from PBMC show their rounded cell morphology. Following a 6-day differentiation period with M-CSF, followed by a 2-day polarization into macrophage subsets, spindle-like cells can be visualized in all subset cultures: (**b**) M0, (**c**) M1, (**d**) M2a, (**e**) M2b, and (**f**) M2c. Images were generated by light microscopy (20x).

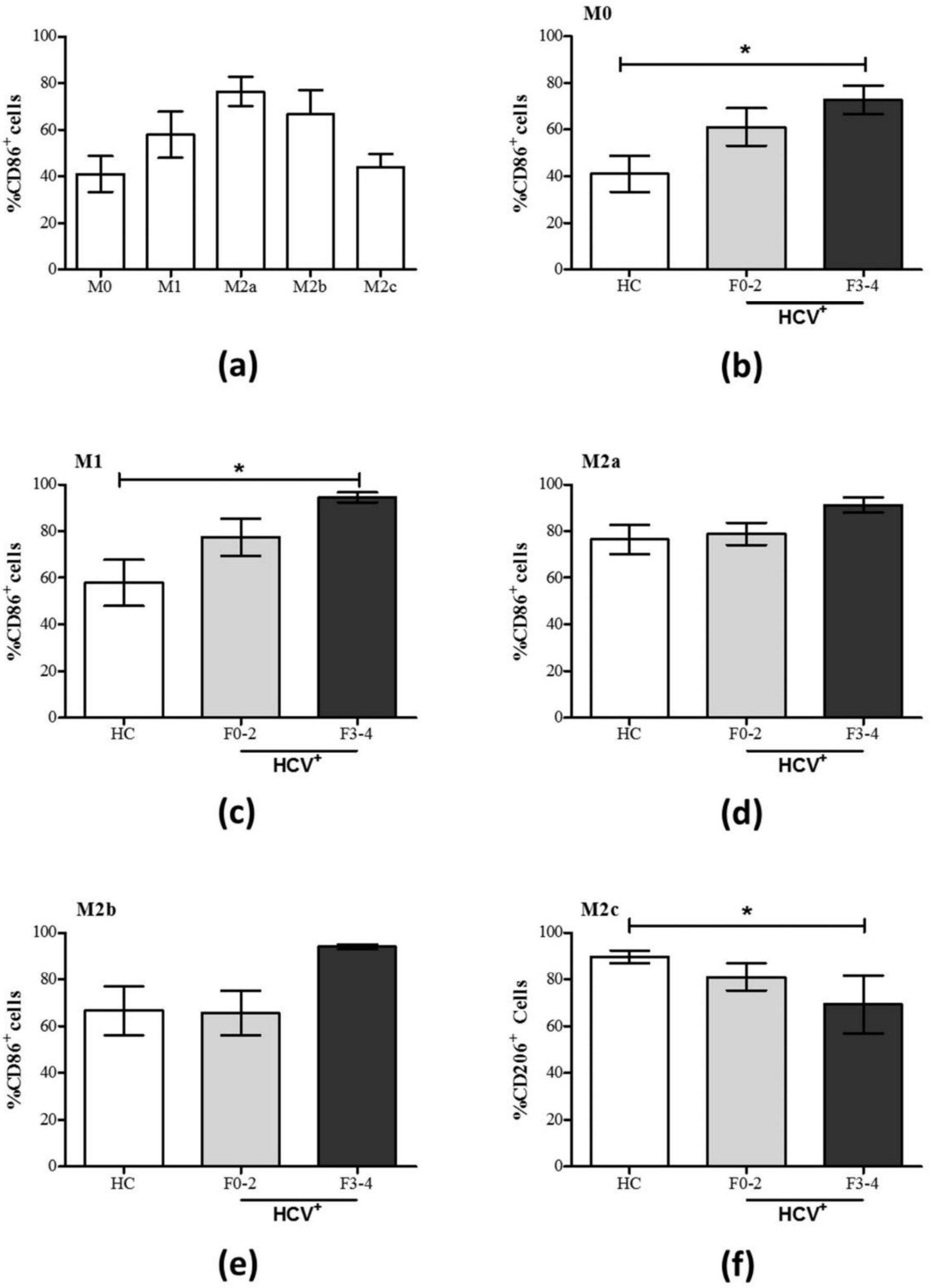

Figure S2. Expression of CD86 in MDM subsets from uninfected controls and HCV-infected individuals. Surface staining of macrophage subsets from healthy controls (HC, n = 7), early fibrosis (F0-2, n = 8), and advanced fibrosis (F3-4, n = 4) was performed and analyzed using flow cytometry. **(a)** The proportion (%) of $CD86^+$ cells across all macrophage subsets from healthy individuals is shown. Changes in %CD86 expression in HCV-infected individuals with minimal (F0-2, n = 9) or advanced liver fibrosis (F3-4, n = 4) are shown for the (**b**) M0 and (**c**) M1 subsets. The remaining subsets are summarized in figures (**d**) M2a, (**e**) M2b, and (**f**) M2c. Statistical significance was determined in healthy controls by one-way, paired Student's *t*-tests, and significance among HCV-infected groups was determined by a one-way ANOVA ($p \leq 0.05$). Significant *p*-values are indicated with an asterisk "*".

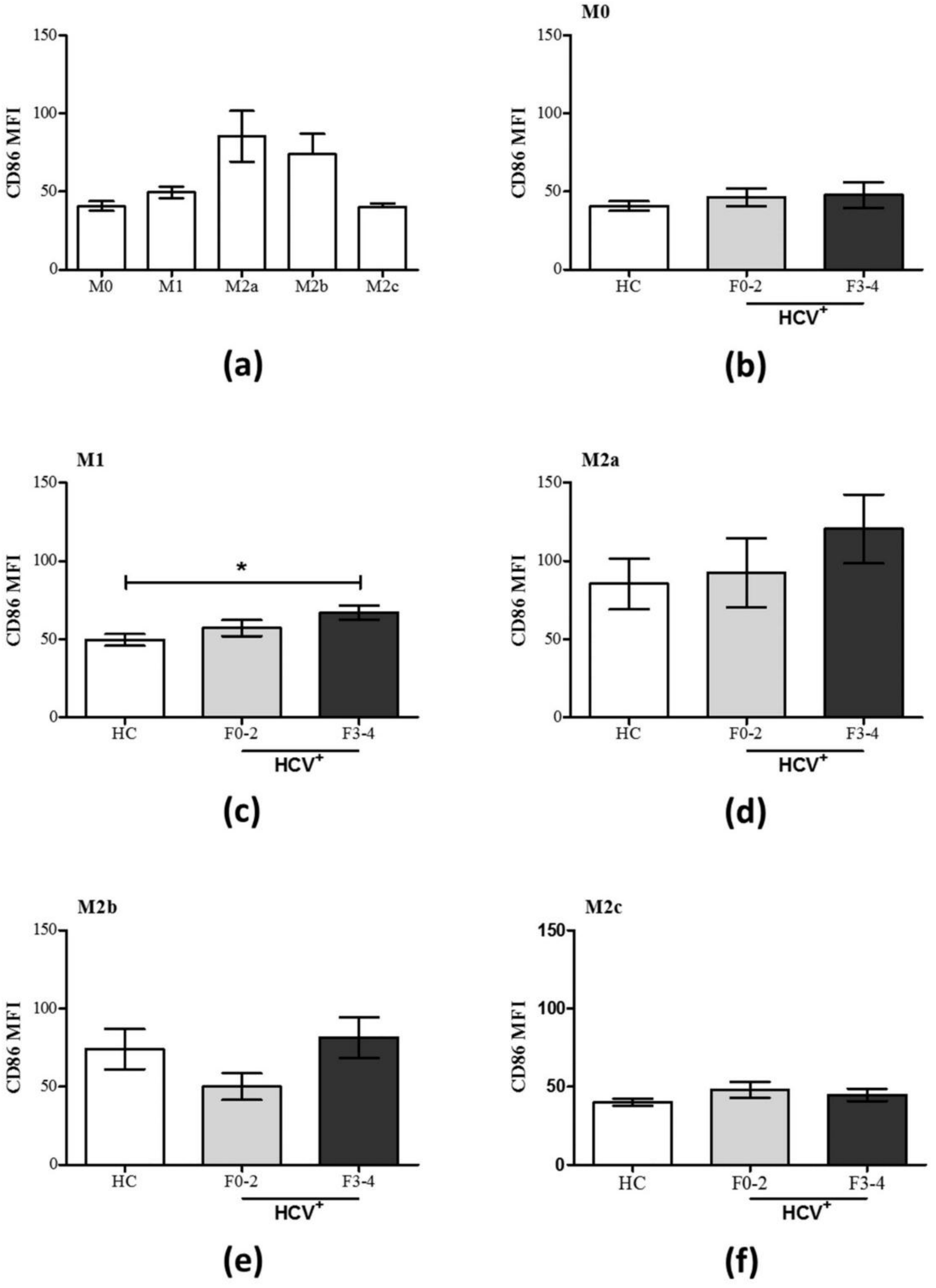

Figure S3. Mean fluorescence intensity of CD86 expression on macrophage subsets from uninfected controls and HCV-infected individuals. Surface staining of macrophage subsets from healthy controls (HC, n = 7), early fibrosis (F0-2, n = 8), and advanced fibrosis (F3-4, n = 4) was performed and analyzed using flow cytometry. (**a**) The mean fluorescence intensity (MFI) of CD86 expression across all macrophage subsets from healthy individuals is shown. Expression of CD86 is also shown for each subset in HCV-infected individuals with early or advanced fibrosis in figures (**b**) M0, (**c**) M1, (**d**) M2a, (**e**) M2b, and (**f**) M2c. Statistical significance was determined in healthy controls by one-way, paired Student's *t*-tests, and significance among HCV-infected groups was determined by a one-way ANOVA ($p \leq 0.05$). Significant *p*-values are indicated with an asterisk "*".

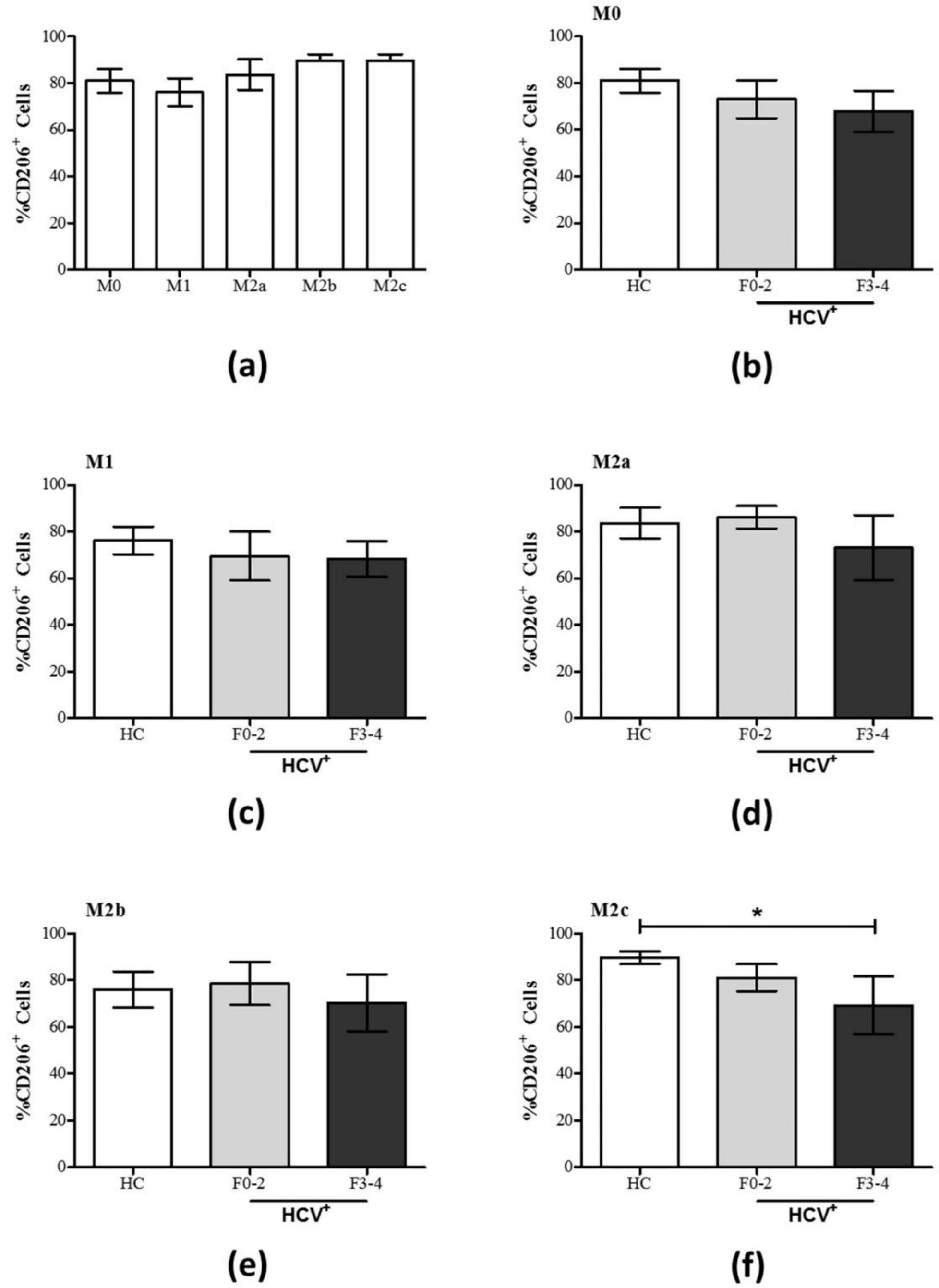

Figure S4. Expression of CD206 on macrophage subsets from uninfected controls and HCV-infected individuals. The expression of CD206 was evaluated on macrophage subsets from healthy controls (HC, n = 9) and HCV-infected individuals with minimal liver fibrosis (F0-2, n = 4) or advanced liver fibrosis (F3-4, n = 4) by flow cytometry. The proportions (%) of $CD206^{+}$ cells (**a**) across all subsets in healthy controls are shown, as well as in the following macrophage subsets for HCV-infected individuals: (**b**) M0, (**c**) M1, (**d**) M2a, (**e**) M2b, and (**f**) M2c**.** Statistical significance was determined in healthy controls by one-way, paired Student's *t*-tests, and significance among HCV-infected groups was determined by a one-way ANOVA ($p \leq 0.05$). Significant *p*-values are indicated with an asterisk "*".

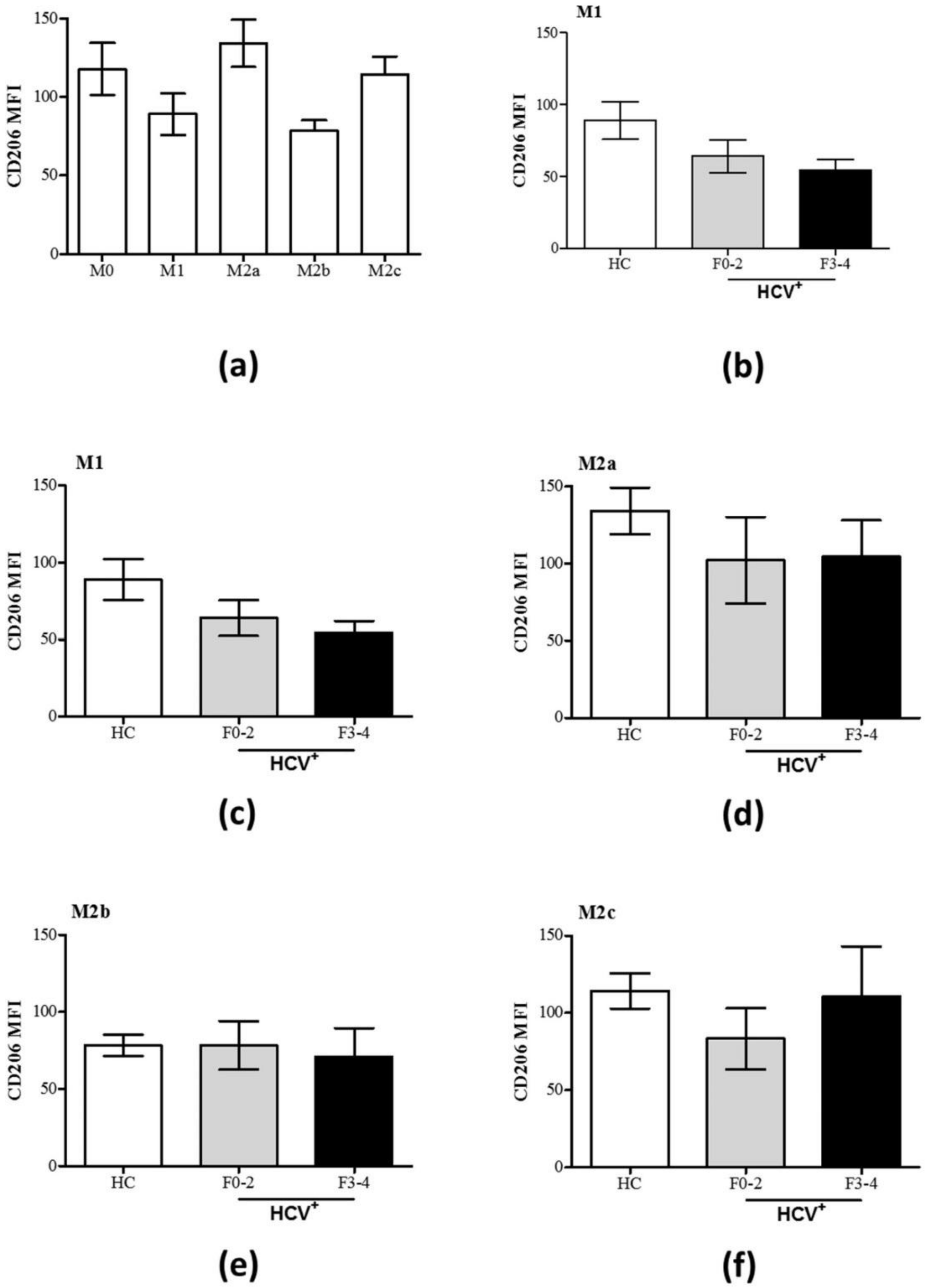

Figure S5. Mean fluorescence intensity of CD206 expression on macrophage subsets from uninfected controls and HCV-infected individuals. The expression of CD206 was evaluated on macrophage subsets from healthy controls (HC, n = 9) and HCV-infected individuals with minimal liver fibrosis (F0-2, n = 4) or advanced liver fibrosis (F3-4, n = 4) by flow cytometry. The mean fluorescence intensity of CD206^{+} cells (**a**) across all subsets in healthy controls are shown, as well as in the following macrophage subsets for HCV-infected individuals: (**b**) M0, (**c**) M1, (**d**) M2a, (**e**) M2b, and (**f**) M2c**.**

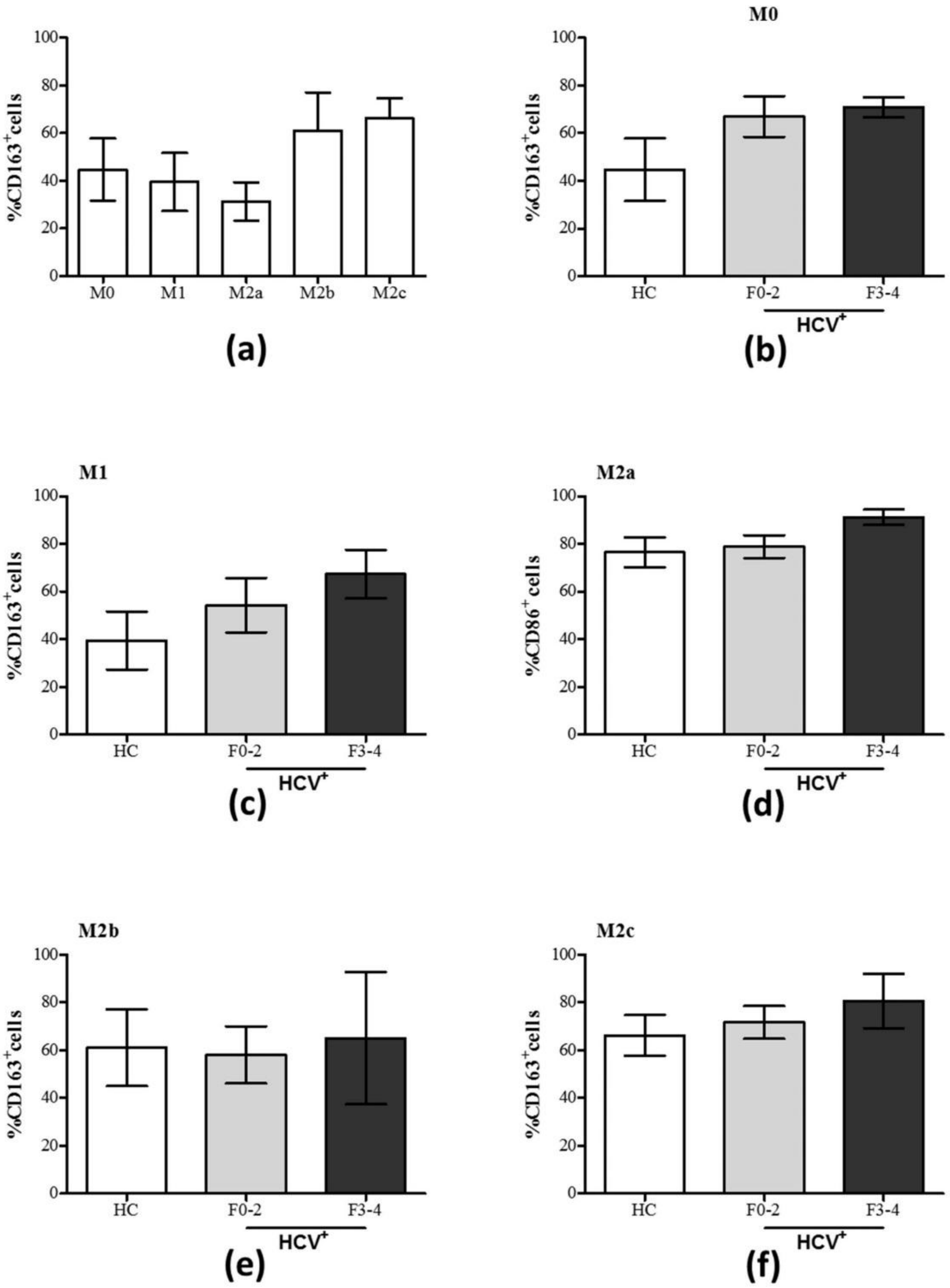

Figure S6. Expression of CD163 on macrophage subsets from uninfected controls and HCV-infected individuals. The expression of CD163 was evaluated on macrophage subsets from healthy controls (HC, n = 9) and HCV-infected individuals with minimal liver fibrosis (F0-2, n = 4) or advanced liver fibrosis (F3-4, n = 4) by flow cytometry. The proportions (%) of $CD163^{+}$ cells (**a**) across all subsets in healthy controls are shown, as well as in the following macrophage subsets for HCV-infected individuals: (**b**) M0, (**c**) M1, (**d**) M2a, (**d**) M2b, and (**f**) M2c.

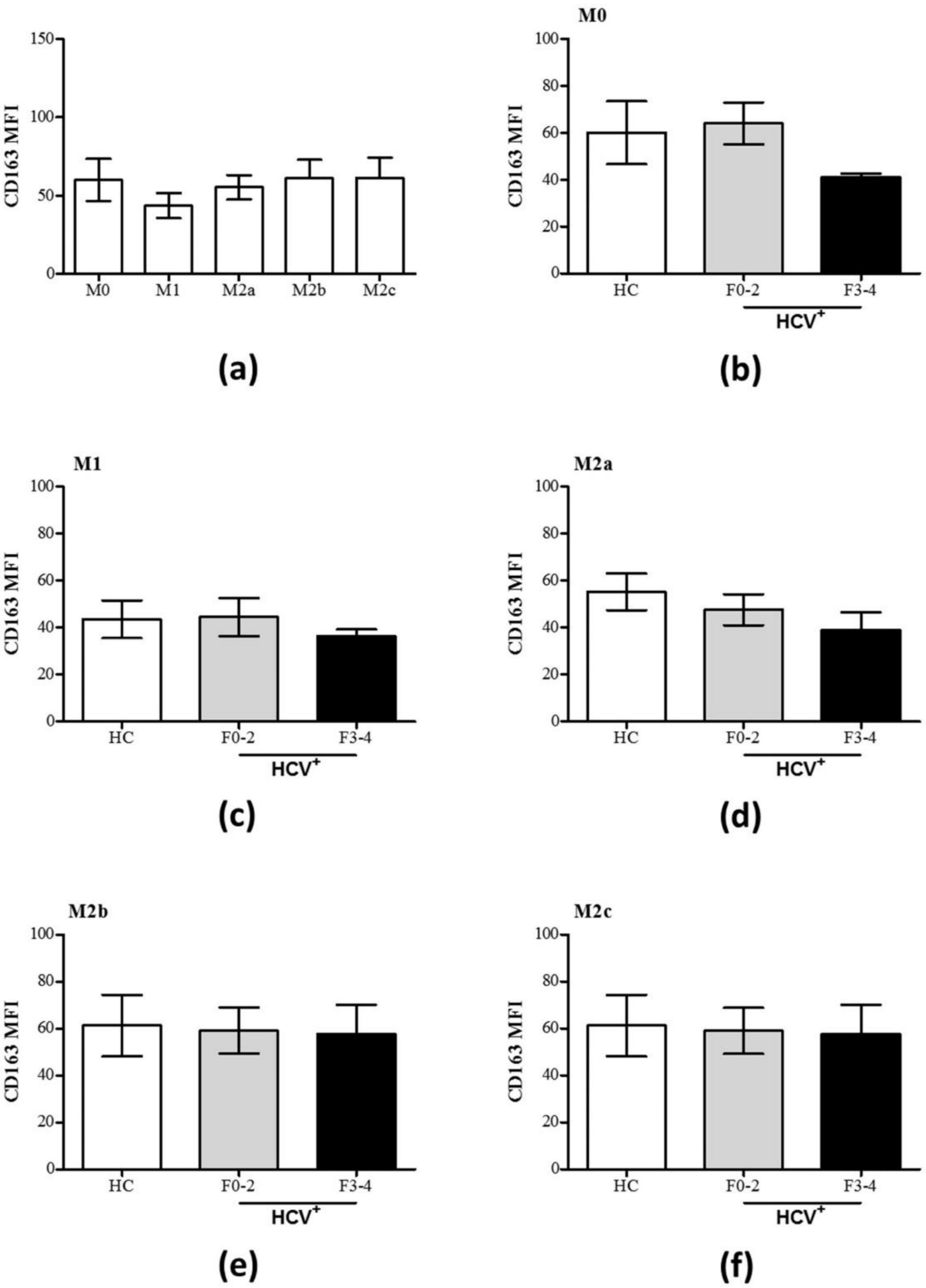

Figure S7. Mean fluorescence intensity of CD163 expression on macrophage subsets from uninfected controls and HCV-infected individuals. The expression of CD163 was evaluated on macrophage subsets from healthy controls (HC, n = 9) and HCV-infected individuals with minimal liver fibrosis (F0-2, n = 4) or advanced liver fibrosis (F3-4, n = 4) by flow cytometry. The mean fluorescence intensity (MFI) of $CD163^{+}$ cells (**a**) across all subsets in healthy controls are shown, as well as in the following macrophage subsets for HCV-infected individuals: (**b**) M0, (**c**) M1, (**d**) M2a, (**d**) M2b, and (**f**) M2c.
